# Does anastomotic technique affects the recurrence rate of Crohn's disease after ileocolic resection?

**DOI:** 10.1016/j.amsu.2021.01.027

**Published:** 2021-01-19

**Authors:** Rashid Ibrahim, Sabry Abounozha, Adel Kheder, Awad Alawad

**Affiliations:** a University Hospitals Plymouth NHS Trust, Plymouth, UK; b Northumbria Healthcare NHS Foundation Trust, Northumbria, UK; c University Hospital Southampton NHS Foundation Trust, Southampton, UK; d University Hospital of Wales, Cardiff, UK

**Keywords:** Anastomosis, Crohn's, Hands sewn, Ileocolic, Kono-S, Staplers

## Abstract

A best evidence topic has been constructed using a described protocol. The three-part question addressed was: does the anastomotic technique after ileocolic resection affects the recurrence rate in patients with Crohn's disease? Using OVID interface and PubMed interface, 16 articles were found; out of this 6 studies were deemed to be suitable to answer the question. The outcomes assessed were anastomotic recurrence rate. The best evidence showed that Kono-S ileocolic anastomotic technique is associated with significantly low recurrence rate in patient with Crohn's disease.

## Highlights

• Kono-S ileocolic anastomotic technique.
• Low recurrence rate.
• Crohn's disease.

## 1 Introduction

This BET was designed using a framework outlined by the International Journal of Surgery [1]. This format was used because a preliminary literature search suggested that the available evidence is of insufficient quality to perform a meaningful meta-analysis. A BET provides evidence-based answers to common clinical questions, using a systematic approach of reviewing the literature.

## 2 Clinical scenario

A colorectal surgical trainee is about to consent a 25-year-old male with recurrent Crohn's disease (CD) after previous ileocecal resection done 5 years ago, for an ileocolic resection. The patient is very concern about the risk of another recurrence, and he is wondering which anastomotic technique is associated with the least recurrence rate?

## 3 Three-part question

Does [the anastomotic technique after ileocolic resection] affects [the recurrence rate] in patients with [Crohn's disease]?

## 4 Search strategy

### 4.1 Embase 1974 to October 2020 using the OVID interface

[Crohn's disease] AND [ileocolic OR ileocolonic] AND [stapler OR staplers OR stapled] AND [hands Sewn OR hand Sewn] AND [anastomotic OR anastomosis] AND [recurrence].

### 4.2 Medline using the PubMed interface

[Crohn's disease] AND [ileocolic OR ileocolonic] AND [stapler OR staplers OR stapled] AND [hands Sewn OR hand Sewn] AND [anastomotic OR anastomosis] AND [recurrence].

The results were limited to English articles and human studies.

## 5 Search outcome

A total of 17 papers were found using both search engines. Out of these 7 papers were excluded because they were irrelevant based on the titles and or the abstracts. Ten full-text articles were screened and assessed for eligibility. From these, six papers were identified to provide the best evidence to answer the question. The anastomotic recurrence is defined as the postoperative need for endoscopic dilatation of the ileocolic anastomosis or neoterminal ileal resection [2].

## 6 Result

see Table 1.

**Table 1:** Search results

Author, date of publication, journal and country	Study type and level of evidence	Patient group + demographics	Outcomes Follow up	Key results	Additional comments
Gaetano Luglio et al. 2020 Ann Surg Italy	Randomized controlled trial level II	Total of 79 ileocolic CD patients were randomized in two groups: Group 1:(Kono-S) = (36) patients, mean age (34), male: female 18:18 Group2:Conventional stapled group (43) patients, mean age (43), male: female 22:21	Primary endpoint: Endoscopic recurrence (ER) Mean 24 periods of follow UP	(ER) rate was: Group1 = 18% Group2 = 30.2% (P 0.04) Statically significant low recurrence rate among (Kono-S) group	-Single centre, -Small sample size, -Short period of follow up -Impossible to blind the endoscopists to each group
Toru Kono et al. 2015 JGastrointest Surg Japan + USA	Retrospective Cohort Study, level III	Total of 187 patients Who underwent (Kono-S) anastomosis Collected from two countries 1.Japan group (144), median age 31, male: female 3:1 2. USA group (43) median age 32, male: female 1:1	Primary endpoint: endoscopic recurrence (ER) Median follow-up was 65 months (43–138) months in group jaban and 32 months (range, 12–44 months) in group US	Only 2 patients out of 187 0.01% had endoscopic recurrence	-Multicentre (international), -Large sample size over all -non-randomized -Retrospective study -Small sample size and shorter follow-up among US group.
Robin S. McLeod, et al. Dis Colon Rectum 2009 Canada.	Multicenter, Randomized, Controlled Trial levelII	A total of 170 patients underwent ileocolonic resection Group1: 84 patients had stapled functional end-to-endAnastomosis, male: female 30:54, mean age 40.3 Group2: 86patients underwent sutured end-to-end anastomosis, male: female 32:54, mean age 38.2	Anastomotic recurrence Confirmed by: Colonoscopy Mean follow-up of 11.9 months	Endoscopic recurrence rate was: Group1: 37.9% Group2: 42.5% (P = 0.55). Difference is not statistically significant	Short period of follow up
Masato Kusunoki et al. 1998 Dig Surg Japan	Randomized controlled trial level II	Total 68 patients Randomized into: Group 1: 32 Stapled functional end-to-end anastomosis, median age 27, male: female 18:9 Group 2: 36 Hand-sewn end to end anastomosis, median age 30.5, male: female 28:8	Anastomotic recurrence Confirmed by: surgery median follow-up period 4.5 years.	Group 1 = 3 patients (0.93%) patients Group 2 = 4 patients (0.11%) patients Difference is not statistically significant	-Single centre, -Small sample size -Only the recurrence that necessitated surrey were studied
Mufioz-Jufirez et al. Dis Colon Rectum 2001 USA	Case-control comparative analysis level III	A total of 138 patients: Group 1: 69 with wide-lumen stapled anastomosis, male: female 34:35, mean age 39.1 group 2: 69 with conventional sutured end-to-end anastomosis male: female 34:35, mean age 38.1	Anastomotic recurrence Confirmed by: surgery median follow group 1: 46 (range, 25–139) months Group2: 70 (range, 13–140) months	Group1 = 11% Group2 = 20% (P = 0.017) Statically significant low recurrence rate among stapled group	-Single centre, -Large sample size, -Retrospective - Shorter follow-up among stapled group. -No randomization -Only the recurrence that necessitated surrey were studied
Yamamoto et al. Scand J Gastroenterolology 1999 UK	Retrospective cohort study, level III	123 patients underwent ileocolonic resection Group 1: 45 patients had stapled functional end-to-end, anastomosis mean age 40, male: female 21:19 Group2: 78 underwent sutured end-to-end anastomosis Mean age 36, male to female 35:43	Anastomotic recurrence Confirmed by: surgery Median follow up: Group 1:34 months Group 2: 92 months	Group 1 = 3% Group 2 = 24% P = 0.007 Statically significant low recurrence rate among stapled group	-Single centre, -Large sample size, -Retrospective -non-randomized - Shorter follow-up among stapled group. -Including only patients with recurrence necessitating surgery

Author, date of publication, journal and country	Study type and level of evidence	Patient group + demographics	Outcomes Follow up	Key results	Additional comments
Gaetano Luglio et al. 2020 Ann Surg Italy	Randomized controlled trial level II	Total of 79 ileocolic CD patients were randomized in two groups: Group 1:(Kono-S) = (36) patients, mean age (34), male: female 18:18 Group2:Conventional stapled group (43) patients, mean age (43), male: female 22:21	Primary endpoint: Endoscopic recurrence (ER) Mean 24 periods of follow UP	(ER) rate was: Group1 = 18% Group2 = 30.2% (P 0.04) Statically significant low recurrence rate among (Kono-S) group	-Single centre, -Small sample size, -Short period of follow up -Impossible to blind the endoscopists to each group
Toru Kono et al. 2015 JGastrointest Surg Japan + USA	Retrospective Cohort Study, level III	Total of 187 patients Who underwent (Kono-S) anastomosis Collected from two countries 1.Japan group (144), median age 31, male: female 3:1 2. USA group (43) median age 32, male: female 1:1	Primary endpoint: endoscopic recurrence (ER) Median follow-up was 65 months (43–138) months in group jaban and 32 months (range, 12–44 months) in group US	Only 2 patients out of 187 0.01% had endoscopic recurrence	-Multicentre (international), -Large sample size over all -non-randomized -Retrospective study -Small sample size and shorter follow-up among US group.
Robin S. McLeod, et al. Dis Colon Rectum 2009 Canada.	Multicenter, Randomized, Controlled Trial levelII	A total of 170 patients underwent ileocolonic resection Group1: 84 patients had stapled functional end-to-endAnastomosis, male: female 30:54, mean age 40.3 Group2: 86patients underwent sutured end-to-end anastomosis, male: female 32:54, mean age 38.2	Anastomotic recurrence Confirmed by: Colonoscopy Mean follow-up of 11.9 months	Endoscopic recurrence rate was: Group1: 37.9% Group2: 42.5% (P = 0.55). Difference is not statistically significant	Short period of follow up
Masato Kusunoki et al. 1998 Dig Surg Japan	Randomized controlled trial level II	Total 68 patients Randomized into: Group 1: 32 Stapled functional end-to-end anastomosis, median age 27, male: female 18:9 Group 2: 36 Hand-sewn end to end anastomosis, median age 30.5, male: female 28:8	Anastomotic recurrence Confirmed by: surgery median follow-up period 4.5 years.	Group 1 = 3 patients (0.93%) patients Group 2 = 4 patients (0.11%) patients Difference is not statistically significant	-Single centre, -Small sample size -Only the recurrence that necessitated surrey were studied
Mufioz-Jufirez et al. Dis Colon Rectum 2001 USA	Case-control comparative analysis level III	A total of 138 patients: Group 1: 69 with wide-lumen stapled anastomosis, male: female 34:35, mean age 39.1 group 2: 69 with conventional sutured end-to-end anastomosis male: female 34:35, mean age 38.1	Anastomotic recurrence Confirmed by: surgery median follow group 1: 46 (range, 25–139) months Group2: 70 (range, 13–140) months	Group1 = 11% Group2 = 20% (P = 0.017) Statically significant low recurrence rate among stapled group	-Single centre, -Large sample size, -Retrospective - Shorter follow-up among stapled group. -No randomization -Only the recurrence that necessitated surrey were studied
Yamamoto et al. Scand J Gastroenterolology 1999 UK	Retrospective cohort study, level III	123 patients underwent ileocolonic resection Group 1: 45 patients had stapled functional end-to-end, anastomosis mean age 40, male: female 21:19 Group2: 78 underwent sutured end-to-end anastomosis Mean age 36, male to female 35:43	Anastomotic recurrence Confirmed by: surgery Median follow up: Group 1:34 months Group 2: 92 months	Group 1 = 3% Group 2 = 24% P = 0.007 Statically significant low recurrence rate among stapled group	-Single centre, -Large sample size, -Retrospective -non-randomized - Shorter follow-up among stapled group. -Including only patients with recurrence necessitating surgery

## 7 Discussion

It is well known that surgical resection and anastomosis is an effective treatment method in complicated ileocecal CD, the main long term consequences of this approach are postoperative recurrences that usually occurs in the preanastomotic area in around 90%. This information might raise the possibility that the anastomotic technique has a role to do in the development of anastomotic recurrence [3].

In this article, we have reviewed the best evince studies which compared the two most common modalities of ileocolic anastomotic techniques in ileocecal CD which are: Stapled functional end-to-end anastomosis and Hand-sewn end to end anastomotic technique in order to assess their relation to the post-operative anastomotic recurrence.

Two studies in our review showed statistically Signficant low incidence of anastomotic recurrence among the stapled group which were conducted by Yamamoto et al. [4] in 1998and Mufioz-Jufirez et al. [5] in 2001. However, there are some limitation to these 2 articles such as single centre, retrospective review which is not without biases and relatively shorter period of follow up among the stapled group.

In contrast, both Masato Kusunoki et al. [6] in 1998 and Robin S. McLeod et al. [3] in 2009 conducted randomised control trials (RCTs) which showed no statistically significant difference in a term of recurrence among the 2 anastomotic techniques, although they are RCTs, there are still some limitations such as short period of follow up, single centre, and small sample size.

In September 2003, Kono et al. at the Asahikawa Medical University Hospital in Japan introduced to the clinical practice a new antimesenteric functional end-to-end hand sewn anastomotic surgical technique to reduce the risk of anastomotic recurrence called (Kono-S anastomosis) [7] this techniques subsequently showed very promising result in reduction of the anastomotic recurrence rate as shown on the large international multicentre retrospective study, conducted by Toru Kono et al., in 2015 [8] although it is retrospective study and it only compare one surgical technique which is Kono-S anastomosis the result showed that the anastomotic recurrence rate was 0.01%, the authors did recommend to conduct a RCT to compare this new technique with the standard stapler techniques which was actually performed recently by Gaetano Luglio et al. [3] in Italy and published recently in 2020, the authors concluded that there is a significant reduction in postoperative anastomotic recurrence among group of patients who underwent Kono-S anastomosis in comparison to the group of patient who underwent conventional stapled technique. Nevertheless, despite some limitation on this RCT such as; single centre, small sample size and relatively short period of follow up, so far the result is promising. The authors do recommend a well-designed large sample size multicentre RCT with longer period of follow up in order to compare this new anastomotic technique with the current conventional techniques.

## 8 Clinical bottom line

According to the above articles, the best evidence showed a significant reduction in postoperative anastomotic recurrence rate, among those patients who had Kono-S antimesenteric functional end-to-end hand sewn ileocolic anastomosis for CD, in comparison to the standard stapled anastomosis. Therefore the authors do recommend performing Kono-S hand sewn anastomosis rather than stapled anastomosis in patient with CD.

Limitation of this review:

1. Small sample size in most articles
2. Shorter period of follow in most articles
3. Some articles relied on the operative finding rather than endoscopy to diagnose recurrence

## Ethical approval

Not applicable.

## Sources of funding

None.

## Author contribution

RI: conducted the literature search and wrote the paper.

SA: assisted in the literature search and Writing of paper.

AK: assisted in writing of paper.

AA: assisted in the literature search, editing of Writing.

## Consent

Not Applicable.

## Registration of research studies

1. Name of the registry: not applicable.

2. Unique Identifying number or registration ID:

3. Hyperlink to your specific registration (must be publicly accessible and will be checked):

## Guarantor

Rashid Ibrahim (RI)1*, Sabry Abounozha (SA)2, Adel Kheder (AK)3, Talal Alshahri4.

## Declaration of competing interest

None.
